# Analysis on the Live Coral Cover around Weizhou Island Using MODIS Data

**DOI:** 10.3390/s19194309

**Published:** 2019-10-04

**Authors:** Rongyong Huang, Huiya Zhang, Kefu Yu

**Affiliations:** 1 Guangxi Laboratory on the Study of Coral Reefs in the South China Sea, Guangxi University, Nanning 530004, China; rongyonghuang@gxu.edu.cn (R.H.); zhanghuiya_gxu@163.com (H.Z.); 2 Coral Reef Research Centre of China, Guangxi University, Nanning 530004, China; 3 School of Marine Sciences, Guangxi University, Nanning 530004, China; 4 Laboratory for Marine Geology, Qingdao National Laboratory for Marine Science and Technology, Qingdao 266061, China

**Keywords:** live coral cover, MODIS, coral reefs, Weizhou Island, South China Sea

## Abstract

Coral reefs are important as they can help to maintain ecological balance, biological resources, and species diversity on earth. However, they are globally threatened by human activities and climate change. As live coral cover (LCC) is regarded as an important measure of the health of coral reefs, analysis on LCC change associated with environmental parameters, such as chlorophyll-a concentration (Chl-a), sea surface temperature (SST), and photosynthetically active radiation (PAR), is of great value. Research on this front would help us comprehend the changes in coral reefs induced by human activities and global changes. Instead of using spasmodically in-field-measured environmental parameters, in this study, we chose to combine the successive Chl-a, SST, and PAR products of the Moderate Resolution Imaging Spectroradiometer (MODIS) with historic LCC records to establish an empirical relationship using nonlinear optimization. Thereafter, the established relationship was further used to discuss some possible developments of LCCs. According to the experiments, we concluded that the degradation of the LCC around Weizhou Island may be mainly caused by human-activity-caused eutrophication. Besides, we also showed that even if the Chl-a and the PAR can keep constant with current average levels, the corals around Weizhou Island may still be in a risk of disappearing between 2120–2140 as the SST continues to rise.

## 1. Introduction

Coral reefs are called the “rainforests of the sea” as they are among the most biodiversity-rich ecosystems in the ocean. They can help to maintain the ecological balance, biological resources, and species diversity on Earth [1]. However, coral reefs are globally threatened owing to the decline in the live coral cover (LCC) [2], where LCC is regarded to be one of the most important measure of coral reef health [3]. To help protect and manage coral reefs, comprehending the knowledge of the impacts of the environment on coral reef ecological status is necessary.

On-site measurements of the environment parameters are usually discontinuous; hence, they are inconvenient for long-term analysis of coral reefs. Fortunately, remote sensing can successively record some coral reef environmental parameters and is thus considered as a powerful tool for research on coral reefs. In the last few decades, satellite assessments mainly focused on sea surface temperature (SST) for understanding the stress on coral reefs [4]. Although high temperature has proven to be one of the main causes of coral bleaching [5], other remote-sensed environmental parameters may also cause coral bleaching, resulting in changes to LCCs [6,7]. Therefore, except for SST, other environmental parameters [8,9,10] such as chlorophyll-a concentration (Chl-a) and photosynthetically active radiation (PAR) should also be combined for detailed comprehension on the responses of coral reefs to the environments. Such research has partly been conducted by some authors [9,10], but they ignored not only the time gaps between the changes of the environmental parameters and the responses of the LCCs, but also the influences of the LCC base on current LCCs.

Many authors [11,12,13,14] have shown that long-term human activities, such as marine engineering, sewage discharge, tourism, etc., can result in degradation of the corals, and Zhao et al. [15] noted that human activities rather than global warming were the primary causes for the degradation of the corals in Luhuitou fringing reef (Hainan). On the other hand, Chl-a concentration usually increases with nutrient concentration [16], while increases in nutrient concentration may be caused by human activities. Further, high PAR may interact with high SST by interfering with the photosystems of the symbiotic zooxanthellae in the corals to exacerbate coral bleaching and resultant mortality.

In other words, SST, Chl-a, and PAR can be regarded as three significant factors to the changes of the LCCs. Coincidentally, a long series of SST, Chl-a, and PAR can be obtained easily from the Moderate Resolution Imaging Spectroradiometer (MODIS) products. Therefore, this paper attempted to model the LCCs around Weizhou Island with respect to the LCC base and the SST, Chl-a, and PAR of MODIS. It considers the time gaps between the changes of the coral reefs and environmental parameters. In addition, the model was further used to discuss the development of the LCCs with respect to an increase in SST. This has rarely been taken into consideration, so is regarded to be a creative application of remote sensing on the research on coral reefs.

## 2. Materials and Methods

### 2.1. Study Area and Experimental Data

**Figure 1:**
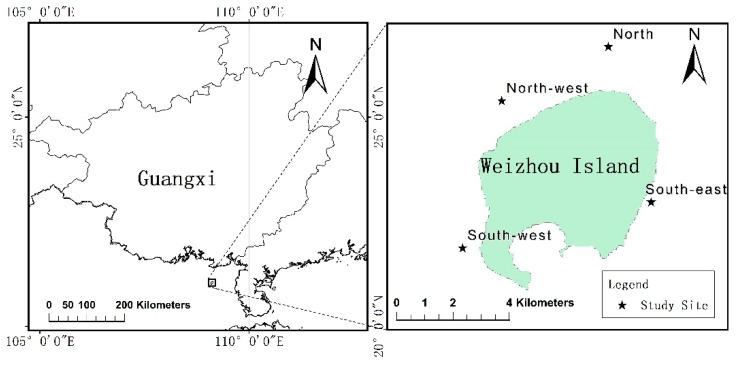
Position of Weizhou Island and the study sites.

Weizhou Island is the largest volcanic island in the Beibu Gulf of the South China Sea (SCS), which is located to the south of Beihai, as shown in Figure 1. According to Wang et al. [16], the annual average SST and sea surface salinity (SSS) are approximately 24.55 °C and 31.9‰, respectively, making it ideal for coral growth and reproduction. According to Wang et al. [16] and Wang and Li [17], the main substrates around Weizhou Island are rock, reef patch, sand and gravel. Sand and gravel are mainly distributed in the north of Weizhou Island, while rocks are distributed in the east and the south-east. In addition, as Weizhou Island is located in a relatively high-latitude area and is heavily influenced by anthropogenic activity, coral reefs have long been the focus of studies that analyze the response of coral reefs to global warming and human disturbance [16,18]. According to Yu et al. [14], for Weizhou Island, *Porites lutea* was the most dominant coral species, *Favites halicora* and *Leptastrea purpurea* were the co-dominant species; Faviidae was the most dominant family followed by Poritidae (According to Budd et al. [19], Faviidae is now restricted to the Atlantic, most Indo-Pacific species previously assigned to this family are now placed in the Merulinidae. However, Faviidae has been used in documents on coral reefs in SCS, e.g., Huang et al. [20], Zhao et al. [21,22], and Yu et al. [14], etc. Hence, this paper continues to use the Faviidae family to show the dominant family of corals around Weizhou Island). Weizhou Island was usually selected as the study area for our experiments on remote sensing of coral reefs, i.e., the bathymetry of the coral reefs [23] and the retrieval of LCCs [14,24]. Similarly, Weizhou Island was chosen as the study area for this study.

**Figure 2:**
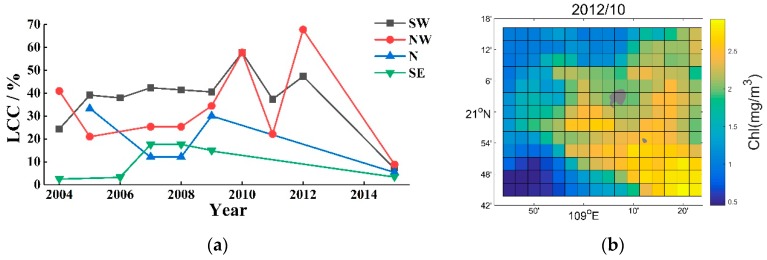

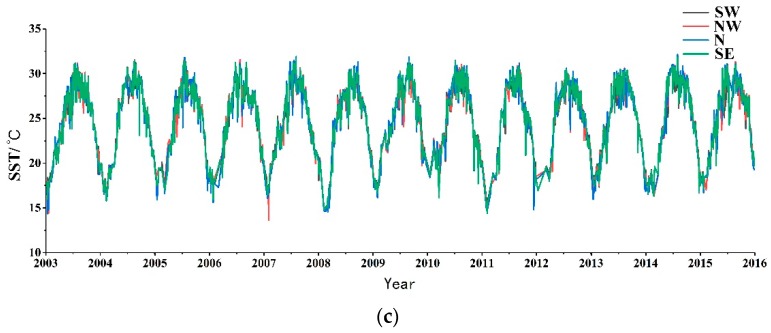
Experimental data: (**a**) Live coral cover (LCC) records from 2004 to 2015; (**b**) average monthly sea surface temperature (SST) around Weizhou Island during October 2012; (**c**) daily SST of the study sites from 2003 to 2015. SW: South-west; NW: North-west; N: North; SE: South-east.

The required LCC data came from historic documents and our in-field investigation. Chen et al. [25] provided the LCCs of the south-west (SW, 2004–2012), north-west (NW, 2004–2005 and 2009–2012), and south-east (SE, 2001–2002, 2004, and 2006–2008) of Weizhou Island by using a coral reef health survey procedure called Reef Check [26]. Huang et al. [27] surveyed the status of the coral reef ecosystems around Weizhou Island and provided the LCCs of the SW, north (N) and SE in 2005 by using the video line intercept transect technique [28,29]. Liang et al. [30] surveyed the LCCs of the SW, NW, N, and SE in 2007 and 2008, adopting the line intercept transect method [28,31]. Similar to Liang et al. [30], Wang and Li [17] also illustrated the LCCs around Weizhou Island in 2009. Besides, we have further observed the LCCs around Weizhou Island using the video line intercept transect technique [24,31]. Those LCC records were chosen as the study materials: 1) The LCCs surveyed by ourselves during 2015 and the LCC sequences of continuous multiyear provided by Chen et al. [25] were selected in priority; 2) the LCCs provided by Huang et al. [27], Liang et al. [30], and Wang and Li [17], etc. were then used as the supplements. The selected LCC data are shown in Figure 2a.

The daily SST, Chl-a, and PAR products (Aqua-MODIS level-3, 4-km spatial resolution) from 2003 to 2015 were downloaded from the Ocean Color Web (https://oceandata.sci.gsfc.nasa.gov). The web is supported by the Ocean Biology Processing Group (OBPG) at the Goddard Space Flight Center (GSFC) of National Aeronautics and Space Administration (NASA). The units are in °C, E/m^2^•day, and mg/m³, respectively. The deep-water area adjoined to Weizhou Island was divided into 4 regions according to the study sites. The average SST, Chl-a, and PAR values in those regions were then calculated respectively and used as the daily environmental parameters corresponding to each study site. Taking SST as an example, Figure 2b shows the distribution of the SST in October 2012, and Figure 2c shows the average SST corresponding to the 4 study sites from 2003 to 2015. The spatial and time variation of the SST can be seen from Figure 2b and Figure 2c respectively.

### 2.2. Processing Methods

To begin the analysis, the time moving average of each environmental factor was calculated at first as follows:

(1) $$e{\left(r,x\right)}_{d,y}=\frac{1}{30r}\sum_{i=d-15r+1}^{d+15r}x_{i}$$

where $e{\left(r,x\right)}_{d,y}$ is the time moving average of the *d*-th day in the *y*-th year; r indicates that e is calculated by using the average of the environmental factor values in r months, which is centered at the *d*-th day in *y*-th year; x presents the remote-sensed environmental factor value, i.e., SST, Chl-a, or PAR; $x_{i}$ indicates that environmental factor value is observed on the *i*-th day of the *y*-th year.

To consider the time gaps of the responses of the LCCs to the environmental parameters, the Pearson correlation coefficient (R) with respect to the LCCs and the environmental parameters are calculated as follows:

(2) $$R(x,d,r,t)=\frac{n\sum_{y}e{\left(r,x\right)}_{d,y-t}LCC_{y}-\sum_{y}e{\left(r,x\right)}_{d,y-t}\sum_{y}LCC_{y}}{\sqrt{n\sum_{y}e^{2}{\left(r,x\right)}_{d,y-t}-{\left(\sum_{y}e{\left(r,x\right)}_{d,y-t}\right)}^{2}}\sqrt{n\sum_{y}LCC_{y}^{2}-{\left(LCC_{y}\right)}^{2}}}$$

where t=0,1,2 denotes the year gap between the changes to the LCCs and the environmental factor x, $LCC_{y}$ represents the LCC in the y year, and n denotes the number of LCCs.

For a certain type of environmental factor x, r, d, and t correspond to the maximal R(x,d,r,t) were then chosen to describe the optimal time gap for the response of the LCCs to x in this paper. We signed those optimal r, d, and t values corresponding to x as $r_{x}$, $d_{x}$, and $t_{x}$ respectively. For convenience, $e{\left(r,x\right)}_{d,y}$ corresponded to $r_{x}$, $d_{x}$, and $t_{x}$ was signed as $x_{y-t_{x}}$ in the following, and was utilized as the environmental parameter for further analysis. Such $x_{y-t_{x}}$ calculated by using the optimal time gaps was then used as the environmental indicator in remaining analysis.

**Figure 3:**
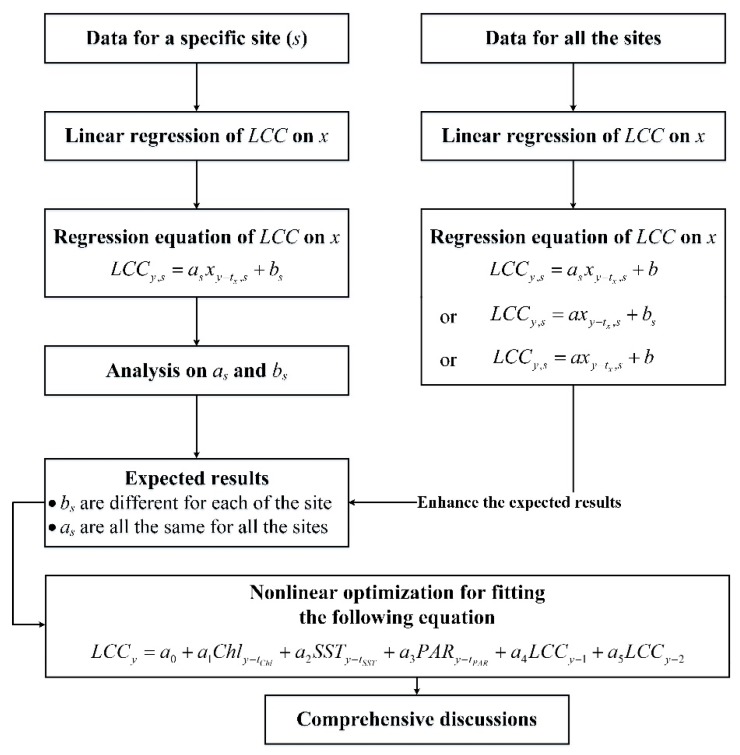
Program for the analysis on the changes of the LCCs associated with environmental parameters. (a. We expected to obtain the result that *a_s_* are all the same for different study sites but *b_s_* are different for each of the study sites by the linear regressions; b. if the expected result was obtained, a nonlinear optimization would be further utilized to form a model described by using the equation).

Thereafter, the empirical relationship of the LCCs around Weizhou Island with respect to SST, Chl-a, and PAR of MODIS was analyzed by using the program shown in Figure 3.

Equation (3) was firstly used to regress the empirical relationship of the LCCs on each specific environmental factor for each of the sites:

(3) $$LCC_{y,s}=a_{s}x_{y-t_{x},s}+b_{s}$$

where s=SW,NW,N,SE, indicated that the study site, $a_{s}$ and $b_{s}$ were solved by using linear regression.

Accordingly, $a_{s}$ and $b_{s}$ for different sites were then compared with each other. We expected to obtain the following results: $a_{s}$ were similar to each other for all the sites, while $b_{s}$ were different for each of the sites. To enhance the result derived from the comparisons, one of the following equations was then selected to do linear regression of the LCCs on each of the environmental factors by using the data of all the sites:

(4) $$LCC_{y,s}=ax_{y-t_{x},s}+b_{s},$$

(5) $$LCC_{y,s}=a_{s}x_{y-t_{x},s}+b,$$

or

(6) $$LCC_{y,s}=ax_{y-t_{x},s}+b.$$

where the actual required equation is chosen as follows: (1) If the comparisons show that $a_{s}$ are similar to each other for all the sites but $b_{s}$ are different for each of the sites, then Equation (4) is chosen; (2) if $a_{s}$ are different for each of the sites but $b_{s}$ are similar to each other for all the sites, then Equation (5) is chosen; (3) if both $a_{s}$ and $b_{s}$ are similar to each other for all the sites, then Equation (6) is chosen.

Finally, we tried to form a more comprehensive model to describe the LCC change associated with environmental parameters. The purpose was to further discuss on the changes of the coral reefs around Weizhou Island with the environments. For example, if the expected results shown in Figure 3 were able to be obtained and enhanced, then Equation (7) can be formed:

(7) $$LCC_{y}=a_{0}+a_{1}Chl_{y-t_{Chl}}+a_{2}SST_{y-t_{SST}}+a_{3}PAR_{y-t_{PAR}}+a_{4}LCC_{y-1}+a_{5}LCC_{y-2},$$

where the items of $a_{4}$ and $a_{5}$ are used to illustrate the impacts of LCC base on current LCCs. Actually, for Equation (7), the differences of the $b_{s}$ in Equation (4) were considered to be caused by the differences of the LCC bases among different sites.

Equation (7) was able to consider the fact that coral populations should be affected not only by the environmental parameters but also the LCC bases. In fact, greater time lags for the LCCs can also be added to the equation, and intuitively, the model should be better for a greater time lag. However, in the following, we will further explain that the number of redundant observations to estimate the coefficients for the experimental data will decrease rapidly as the time lag increases.

The main advantages of Equation (7) were as follows: 1) SST, Chl-a, and PAR can be comprehensively considered to influence on the changes of the LCCs; 2) the time gaps between the changes of the LCCs and the environment factors can also be considered; 3) the LCC bases are introduced by $a_{4}$ and $a_{5}$ items, so the future LCCs can be estimated iteratively by using the LCCs over the past several years and the predictions of the environment parameters.

The disadvantage is that there was a lack of LCC data in some years for each study site. Based on Figure 2a, only 14 pairs of known LCCs and corresponding environmental parameters can be formed and used as constraints to linearly regress Equation (7), as shown in Table 1. However, the equation had 6 unknown coefficients, so the linear regression had only 8 redundant observations. To increase the number of the redundant observations, we further added the constraints formed by the following way to the regression approach: Take 2012 to 2014 for example, the LCC of the SW in 2013 was unknown, so the LCCs of the SW from 2012 to 2014 cannot form a constraint for the linear regression of Equation (7), even though both the LCC in 2012 and in 2014 are known; but as the LCC of the SW in 2011 is known, the LCC in 2013 can be expressed by using Equation (7); this expression can be further substituted into the constraint formed by the LCCs from 2012 to 2014, and then formed a nonlinear constraint. The number of the constraints can be then increased from 14 to 22 by this method, and the number of the redundant observations is then increased to 15. Accordingly, the coefficients of the equation can be then solved by using nonlinear optimization of some software, such as MATLAB and Mathematica, etc.

On other hand, the number of the redundant observations will rapidly decrease as the time lag increases: For example, if the four-year time lag was used, then the number of the redundant observations for our experimental data was reduced to 10. In this paper, we selected two-year time lag to ensure both a relative long time lag and a relative large number of redundant observations.

Finally, we further point out that non-linear optimization was necessary. If linear regression was used instead of the non-linear optimization, we had to discard some LCCs such as the LCC data surveyed in 2015, as shown in Table 1. What is more, when we regressed Equation (7) using the data listed in Table 1, the coefficient of $LCC_{y-1}$ was seen to be negative, and the coefficient of $LCC_{y-2}$ was seen to be positive. This is unreasonable. The reason is $LCC_{y-1}$ should not has a negative impact on the current LCC, as it is a base of the current LCC.

## 3. Results

According to Equation (2), R(x,d,r,t) can be seen as a function of x, r, d, and t. Hence, for a specific type of environmental factor x, we can search r, d, and t that correspond to the maximum of the sum of the absolute R(x,d,r,t) of the four study sites. Those searched values were used to describe the optimal time gap for the LCC responses to x. As a result, we obtained the following results: 1) For Chl-a (x=Chl), $r_{Chl}=4$, $d_{Chl}=235$, and $t_{Chl}=2$, i.e., the average of the Chl-a from May 25 to October 22 in 2 years ago was regarded to have the greatest impact on the changes to the LCCs; 2) for SST (x=SST), $r_{SST}=3$, $d_{SST}=124$, and $t_{SST}=1$, i.e., the average of the SST from the February 21 to May 18 from one ryear ago was regarded to have the greatest impact on the changes to the LCCs; 3) for PAR (x=PAR), $r_{PAR}=3$, $d_{PAR}=211$, and $t_{PAR}=2$, i.e., the average of the PAR from May 16 to August 13 from two years ago was regarded to have the greatest impact on the changes to the LCCs. Environmental indicators ($x_{y-t_{x}}$) calculated by using those optimum parameters were then used in the following sections.

Thereafter, the empirical relationships of the LCCs on each specific environmental factor for each of the sites obtained by using the linear regressions were checked. We observed that most regression coefficients of a single environmental factor are close to each other for different study sites, except for the constant terms, e.g., for $LCC_{y,SW}=-0.22777Chl_{y-2,SW}+0.83685$ (r=−0.74), and $LCC_{y,N}=-0.2137Chl_{y-2,N}+0.66855$ (r=−0.70), –0.22777 was close to –0.2137; for $LCC_{y,SW}=-0.11625SST_{y-1,SW}+3.24287$ (r=−0.60) and $LCC_{y,SE}=-0.11888SST_{y-1,SE}+3.04111$ (r=−0.78), –0.11625 was close to –0.11888. As a result, we guessed that the regression equations of different sites shared a coefficient for a certain environmental factor, i.e., Equation (3) can be updated to Equation (4).

According to the program shown in Figure 3, we should further regress Equation (4). For convenience, we further normalized $LCC_{y,s}$ and $x_{y-t_{x},s}$ as follows:

(8) $$LC{C^{\prime }}_{y}=LCC_{y,s}-\bar{LCC_{y,s}},$$

(9) $${x^{\prime }}_{y-t_{x}}=x_{y-t_{x},s}-\bar{x_{y-t_{x},s}},$$

where $\bar{LCC_{y,s}}$ and $\bar{x_{y-t_{x},s}}$ are the averages of $LCC_{y,s}$ and $x_{y-t_{x},s}$ on the study site s. Then, Equation (4) can be rewritten as:

(10) $$LC{C^{\prime }}_{y}=a{x^{\prime }}_{y-t_{x}}.$$

**Figure 4:**
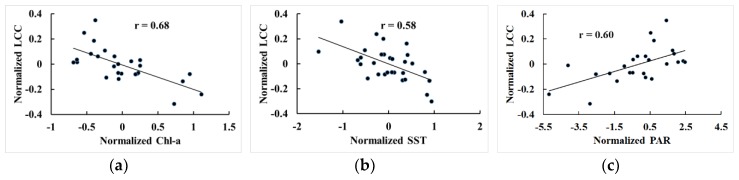
Regression of the normalized chlorophyll-a concentration (Chl-a), SST, and photosynthetically active radiation (PAR) to the normalized LCC: (**a**) $Ch{l^{\prime }}_{y-2}$ v.s. $LC{C^{\prime }}_{y}$; (**b**) $SS{T^{\prime }}_{y-1}$ v.s. $LC{C^{\prime }}_{y}$; (**c**) $PA{R^{\prime }}_{y-2}$ v.s. $LC{C^{\prime }}_{y}$.

Hence linear regression of Equation (4) is equivalent to regressing Equation (10). As shown in Figure 4, the linear regression equation of $LC{C^{\prime }}_{y}$ versus ${x^{\prime }}_{y-t_{x}}$ indicated that the normalized LCC ($LC{C^{\prime }}_{y}$) to a certain extent are proportional to the normalized Chl-a ($Ch{l^{\prime }}_{y-2}$), SST ($SS{T^{\prime }}_{y-1}$), and PAR ($PA{R^{\prime }}_{y-2}$). Thus, it is reasonable to assume that the regression equations of different sites share the coefficient for a single environmental factor except for the constant terms.

In addition, coral populations should be affected not only by the environmental parameters but also the bases. That is, except for Chl-a, SST, and PAR, current LCCs should also depend on LCCs in the previous one, two, or even more years. This may be the reason why the constant terms are different for different study sites. Based on these conjectures and considering the LCCs in the last two years, the current LCCs can be further modelled by combining the remote-sensed environmental parameters with the previous LCCs, as shown in Equation (7). The equation was solved by using the nonlinear optimization of MATLAB, and the result is as follows:

(11) $$LCC_{y}=1.7899-0.1135Chl_{y-2}-0.06792SST_{y-1}+0.00963PAR_{y-2}+0.2542LCC_{y-1}+0.2177LCC_{y-2}.$$

Cross-validation was finally conducted using the leave-one-out (LOO) method to check the performance of model (11). The result demonstrated that the mean relative error (MRE), mean absolute error (MAE), root-mean-square error (RMSE), and maximum absolute error (MAX) was 38%, 0.14, 0.16, and 0.29, respectively. These statistics indicate that Equation (11) is effective for analyzing the change trends of the LCCs around Weizhou Island.

## 4. Discussion

### 4.1. Supplemental Discussion on the Effectiveness

**Figure 5:**
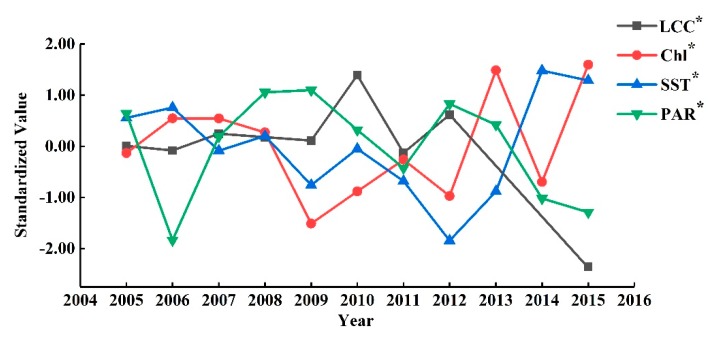
Standardized LCC and corresponding environmental parameters of the SW site.

To visually comprehend the empirical relationship between the environmental parameters and the changes of the LCCs expressed by Equation (11), we took the SW site as an example to plot the LCC, Chl-a, SST, and PAR with respect to the years in Figure 5. In the figure, LCC, $Chl_{y-2}$, $SST_{y-1}$, and $PAR_{y-2}$ are standardized by using the averages and standard deviations as follows:

(12) $$x^{*}=\frac{x-\mu_{x}}{\sigma_{x}},$$

where x represents LCC, $Chl_{y-2}$, $SST_{y-1}$, or $PAR_{y-2}$; $\mu_{x}$ and $\sigma_{x}$ are the corresponding expectation and standard deviation respectively. Such standardizations make those values be in the same order of magnitude, and is convenient for the comparisons.

We observed that the signs of the coefficients of environment parameters in Equation (11) are consistent with Figure 5. From 2005 to 2006, the Chl-a and the SST were both slightly higher than the averages, while the PAR was decreasing. This led to a small decline of the LCC. From 2006 to 2010, the standardized Chl-a had a decreasing trend, the standardized PAR had an increasing trend, and the standardized SST had a slightly decreasing trend. This environment is relatively proper for the growth of the corals, hence the LCC showed an overall increasing trend. From 2010 to 2015, the LCC was significantly decreasing. In this period, the Chl-a and SST were rapidly increasing, while the PAR was overall decreasing. Such observation actually enhanced the effective of Equation (11) for analysis on the LCC change trends.

### 4.2. Discussion on the Responses of the LCCs to the Environments

To compare the impacts of Chl-a, SST, and PAR to the changes of the LCCs, Equation (11) was further transformed to an equivalent expression for standardized $Chl_{y-2}$, $SST_{y-1}$, and $PAR_{y-2}$ (i.e., $Chl_{y-2}^{*}$, $SST_{y-1}^{*}$, and $PAR_{y-2}^{*}$). These standardizations were expressed by Equation (12), where x represents $Chl_{y-2}$, $SST_{y-1}$, or $PAR_{y-2}$ instead. As a result, we found that the coefficients of $Chl_{y-2}^{*}$, $SST_{y-1}^{*}$, and $PAR_{y-2}^{*}$ were –0.05484, –0.04654, and 0.019018 respectively.

The coefficient of standardized $Chl_{y-2}$ was negative and observed to be the largest among the three environmental factors. Actually, Chl-a was regarded to be one of the most important markers of water eutrophication: There was a strong positive correlation between nutrition concentration and Chl-a concentration [32]. In other words, the Chl-a concentration usually increased with nutrient concentration. Many studies have also shown that global coral reef degradation has a close association with the human-activity-caused water eutrophication [33,34,35,36], i.e., increases in nutrition concentration has a negative influence on coral growth. This means that water eutrophication caused by human activities may be the main reason for the degradation of the coral reefs around Weizhou Island.

Shi et al. [11] pointed out that the main causes of decreasing LCC at Luhuitou fringing reef (Hainan) were human activities such as sewage and waste discharge, which lead to eutrophication. Further, Zhao et al. [15] stated that human activities were primarily responsible for LCC declines on Luhuitou fringing reef rather than large-scale coral bleaching caused by global warming. More immediately, Yu et al. [14] illustrated that water eutrophication caused by intensive anthropogenic activities had led to rapid decline of the LCC around Weizhou Island. This greatly enhanced the above analysis, i.e., Chl-a having the greatest influence on LCC suggested that local anthropogenic inputs may be the biggest threat to the coral rather than SST for Weizhou Island.

Specifically, many nutrients such as nitrogen and phosphorus compounds usually inflow into coral reef regions. Such nutrients mainly came from human activities, such as industrial wastewater and domestic sewage discharge, pesticide and fertilizer utilization, and fish farming, etc. They can cause increasing water eutrophication. Furthermore, the increase of water eutrophication reduces the coral calcification and fertilization rates, increases macroalgal abundances [37], and even enhances some diseases progression in corals [38]. The result is that the corals die or their growth slows down. Besides, the water eutrophication may also be able to aggravate the biological erosion of the carbonate skeleton of the coral reefs. For example, Chen et al. [39] pointed out that biological erosion in the south of Weizhou Island was significantly higher than in the north, as the water eutrophication in the south was more serious. According to Huang et al. [27], with the development of tourism, more and more sewage and household refuse were output to the sea water around Weizhou Island. As a result, the nutrition concentration became larger and larger, leading to more algae pollution breaking out. The algae pollution further lead to the degradation of the coral reefs.

Although SST was considered to have greatly impact on global coral growth [5,36,40], our results showed that Chl-a had the greatest influence on the LCCs around Weizhou Island followed by SST and PAR. The reason may be due to the fact that Weizhou Island is a relatively high-latitude coral reef area. According to Han et al. [41], in such a relatively high-latitude coral reef area, the chance of coral cold bleaching is greater than hot bleaching. Therefore, under global warming trends, SST may become not the most significant factor on the changes of the LCCs for Weizhou Island. On the other hand, Chen et al. [42] pointed out that global warming may lead to the decrease of the coral growth rates around Weizhou Island. However, at the same time, their coral growth rate data also showed that the decrease of the coral growth rate or even the bleaching of the corals was able to be recovered, if there were few environmental stresses on the coral reefs. Hence Chen et al. [42] also concluded that the most important factor on large areas of coral degeneration around Weizhou Island may not come from global warming. This is consistent to our results, as it was able to explain why the coefficient of $SST_{y-1}^{*}$ was shown to be smaller than $Chl_{y-2}^{*}$.

Finally, a certain amount of PAR not only plays a role in promoting the coral growth [43], but also makes algae grow better [44]. Hence PAR is able to promote the increase of Chl-a at the same time. Meanwhile, algae and coral are competitive with each other for space and light [45]. In summary, PAR has a negative effect on the reproduction and recovery processes of corals. Due to the canceling of positive and negative effects, the influence of PAR on LCCs is seen to become small, as shown in the coefficient of $PAR_{y-2}^{*}$.

Note that the coefficients of $LCC_{y-1}$ and $LCC_{y-2}$ are shown non-zeroes and positive, so we consider that except for Chl-a, SST, and PAR, current LCCs should be also determined by the previous LCC bases. Furthermore, the effects of the LCC bases are shown in a decreasing trend as the years become longer, i.e., the coefficients of $LCC_{y-2}$ (0.2177) is smaller than that of $LCC_{y-1}$ (0.2542). This is consistent with the intuition.

In summary, current LCC is regarded to be determined by both the LCC bases and the environments. For the environment parameters, Chl-a was shown to have most greatly negative impacts on the coral growth, followed by SST. In contrast, PAR was shown to have a small positive effect on the increase of the LCCs. In other words, SST was considered to greatly impact the degradation of global coral reefs [5,36,40], but it may be different for relatively high-latitude coral reef regions. Human-activity-caused water eutrophication may have become the most significant factor for the degradation of the coral reefs around Weizhou Island.

### 4.3. Discussion on the Changes of the LCCs to the Environments

To understand how the LCCs around Weizhou Island change when the environmental parameters remain constant, we calculated the averages of Chl-a, SST, and PAR from 2003 to 2015 respectively by using the following equation:

(13) $$\bar{x}=\frac{1}{13}\sum_{y=2003}^{2015}e{\left(r_{x},x\right)}_{d_{x},y}.$$

**Figure 6:**
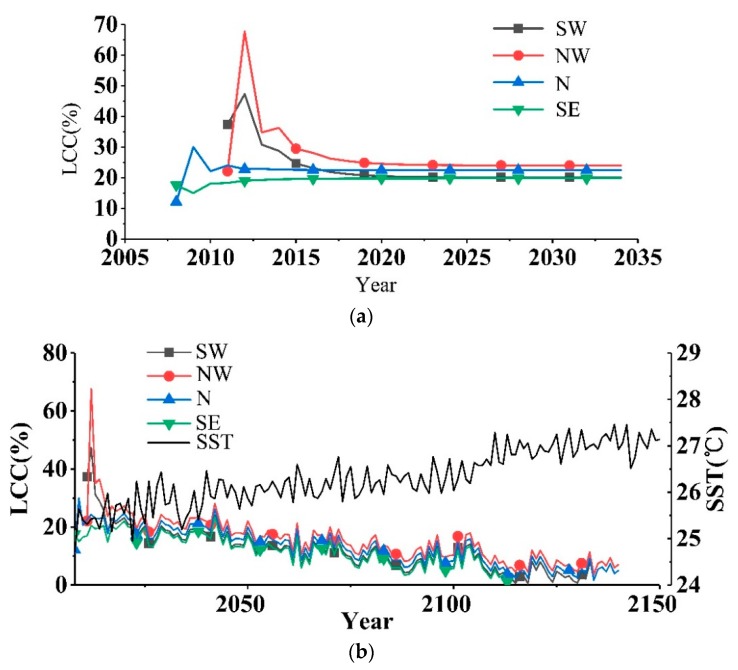
Developments of the LCCs around Weizhou Island under the conditions of (**a**) constant environmental parameters and (**b**) constant environmental parameters except for the raising of SST.

Thereafter, those averaged Chl-a, SST, and PAR, together with the nearest previous two LCCs, were then iteratively substituted into Model (11) and they produced a series of predicted LCCs for future years, as shown in Figure 6a.

It can be seen from Figure 6a that if Chl-a, SST, and PAR stay as per the averaged values, the LCCs tend to be attenuated for SW and NW and slightly increased for N and SW until about 2020. In other words, if the environment stays unchanged over time in current averages, the LCCs around Weizhou Island tend to remain constant. In fact, the constant can be calculated by setting $LCC_{y}=LCC_{y-1}=LCC_{y-2}$ to Equation (11) as follows:

(14) $$LCC=\frac{1.7899-0.1135\bar{Chl}-0.0679\bar{SST}+0.00963\bar{PAR}}{1-0.2542-0.2177}.$$

According to the analysis of Zheng [46] on the temperature data from 1960 to 2011, the rise in the SST of Weizhou Island was 0.0084–0.0159 °C/year, i.e., the degree of the rise of the SST was approximately 0.43–0.83 °C from 1960 to 2011. To further study the changes of the LCCs with respect to the increase in the SST, we simulated the SST to rise randomly from 0.0084 °C to 0.0159 °C every year with Chl-a and PAR being constant at their averages expressed by Equation (13). Furthermore, some random fluctuations were also added to the SST series. The fluctuations were subjected to a normal distribution with 0 °C and 0.15 °C^2^ as its mean and variance, respectively. The variance was selected to be equal to the one estimated by Zhang [47] for Weizhou Island. Such environmental series are then iteratively substituted into Equation (11), as shown in Figure 6b.

The result indicated that the LCCs around Weizhou Island may be degraded year-on-year as the SST rises. It means that although Weizhou Island is a relatively high-latitude coral reef area, being regarded as a coral refugia for the refuge hypothesis [48], LCC degradation may be still inevitable in the case of climate warming. The difference is that the time for the possible disappearance of the coral reefs may be delayed to around 2120–2140, whereas the LCCs in low-latitude areas are predicted to disappear in 20–80 years by some other authors [49,50].

## 5. Conclusions

We combined historic in-field LCC records together with the Chl-a, SST, and PAR series of MODIS to empirically model the relationship of the LCCs around Weizhou Island with respect to Chl-a, SST, and PAR. The model can not only consider the delay of effects of the environmental parameters on the changes to the LCCs but also describe the influences of LCCs of previous years on the current ones. According to the model, instead of the rise of the SST, human-activity-caused water eutrophication may have become the most significant factor for the degradation of the coral reefs around Weizhou Island. Furthermore, the proposed model was used to analyze on the development of the LCCs around Weizhou Island. Its main conclusions are as follows: (1) The LCCs around Weizhou Island tend to remain constant for constant environments; (2) the corals may face the danger of disappearance under current average level of water eutrophication and global warming.

In addition, remote sensing was verified to be a powerful tool for research on coral reef ecosystems. This was deemed as creative. Certainly, there are also some aspects that could be improved in the future for enhancing the model and its performance on the analysis. As the time lag in the experiments was objectively chosen, other time lags should be further compared with each other for searching the best effects. As only three environmental factors were considered, more environmental factors such as particulate organic carbon (POC) product of MODIS should also be considered in future research. As the remote-sensed environmental factors are just the values of the sea surface rather than the ones reaching the corals, some technologies should be specially developed to correct the values of the sea surface to the ones reaching the corals for improving the analysis.

## Figures and Tables

**Table 1:** Pairs of LCC data used as constraints to the linear regression.

Site	Pair ID	Year			LCC (%)		
		*y*	*y-1*	*y-2*	*LCC_y_*	*LCC_y-1_*	*LCC_y-2_*
**South-west**	1	2006	2005	2004	37.9	39.1	24.3
	2	2007	2006	2005	42.3	37.9	39.1
	3	2008	2007	2006	41.4	42.3	37.9
	4	2009	2008	2007	40.5	41.4	42.3
	5	2010	2009	2008	57.7	40.5	41.4
	6	2011	2010	2009	37.32	57.7	40.5
	7	2012	2011	2010	47.3	37.32	57.7
**North-west**	8	2009	2008	2007	34.4	25.3	25.3
	9	2010	2009	2008	57.7	34.4	25.3
	10	2011	2010	2009	22.1	57.7	34.4
	11	2012	2011	2010	67.7	22.1	57.7
**North**	12	2009	2008	2007	30.0	12.21	12.21
**South-east**	13	2008	2007	2006	17.58	17.58	3.3
	14	2009	2008	2007	15.0	17.58	17.58

## References

[1] Liao B., Liu L., Liu C. (2011). Research Status and Prospects of Xuwen Coral Reefs. J. Guangdong Ocean Univ..

[2] Carpenter K.E., Abrar M., Aeby G., Aronson R.B., Banks S., Bruckner A., Chiriboga A., Cortés J., Delbeek J.C., DeVantier L. (2008). One-Third of Reef-Building Corals Face Elevated Extinction Risk from Climate Change and Local Impacts. Sci..

[3] Yu K. (2012). Coral reefs in the South China Sea: Their response to and records on past environmental changes. Sci. Chin. Earth Sci..

[4] Strong A., Liu G., Skirving W., Markeakin C. (2011). NOAA’s Coral Reef Watch program from satellite observations. Ann. GIS.

[5] Lesser M.P. (2011). Coral Bleaching: Causes and Mechanisms. Coral Reefs: An Ecosystem in Transition.

[6] Douglas A.E. (2003). Coral bleaching—how and why?. Mar. Pollut. Bull..

[7] Shaazia M., Scott H., Rajindra M., Ricardo C. (2015). Performance Evaluation of CRW Reef-Scale and Broad-Scale SST-Based Coral Monitoring Products in Fringing Reef Systems of Tobago. Remote Sens.-Basel.

[8] Liu G., Heron S., Eakin C.M., De La Cour J., Geiger E., Skirving W., Burgess T., Strong A. (2014). NOAA Coral Reef Watch’s Next-Generation 5 km Satellite Coral Bleaching Thermal Stress Monitoring. News Int. Soc. Reef Stud..

[9] Maina J., Venus V., McClanahan T.R., Ateweberhan M. (2008). Modelling susceptibility of coral reefs to environmental stress using remote sensing data and GIS models. Ecol. Model..

[10] Barnes B.B., Hallock P., Hu C., Muller-Karger F., Palandro D., Walter C., Zepp R. (2015). Prediction of coral bleaching in the Florida Keys using remotely sensed data. Coral Reefs.

[11] Qi S., Meixia Z., Lingying H., Hongqiang Y., Huiling Z. (2010). Human Activities and Impacts on Coral Reef at the Luhuitou Fringing Reef, Sanya. Trop. Geogr..

[12] Wu Z., Wang D., Ye C., Li Y., Chen M., Chen C. (2012). Variation tendency and analysis of cause of coral in Sanya. Mar. Environ. Sci..

[13] Zhou H., Yao X., Li L., Geng T., Zhang Y. (2017). Scleractinian coral community structure and distribution in the coastal waters surrounding Hainan Island. Biodivers. Sci..

[14] Yu W., Wang W., Yu K., Wang Y., Huang X., Huang R., Liao Z., Xu S., Chen X. (2019). Rapid decline of a relatively high latitude coral assemblage at Weizhou Island, northern South China Sea. Biodivers. Conserv..

[15] Zhao M., Yu K., Qiaomin Z., Shi Q. (2011). Evaluating the sustainability of coral reefs in Sanya of Hainan Island using marine ecological footprint. J. Trop. Oceanogr..

[16] Wang W., Yu K., Wang Y. (2016). A Review on the Research of Coral Reefs in the Weizhou Island, Beibu Gulf. Trop. Geogr..

[17] Wang X., Li G. (2009). The Status and Prospect of Researches on Coral Reef in Weizhou Island. J. Guangxi Academy Sci..

[18] Chen T., Li S., Yu K., Zheng Z., Wang L., Chen T. (2013). Increasing temperature anomalies reduce coral growth in the Weizhou Island, northern South China Sea. Estuarine, Coast. Shelf Sci..

[19] Budd A.F., Fukami H., Smith N.D., Knowlton N. (2012). Taxonomic classification of the reef coral family Mussidae (Cnidaria: Anthozoa: Scleractinia). Zool. J. Linn. Soc..

[20] Huang H., Zhang Y., Lian J., Li X., You F., Yang J., Lei X., Zhang C. (2011). Structure and diversity of scleractinia coral communities along the west seashore of Xuwen County. Biodivers. Sci..

[21] Zhao M., Yu K., Zhang Q., Shi Q., Price G.J. (2012). Long-term Decline of a Fringing Coral Reef in the Northern South China Sea. J. Coast. Res..

[22] Zhao M., Yu K., Shi Q., Yang H., Riegl B., Zhang Q., Yan H., Chen T., Liu G., Lin Z. (2017). Comparison of coral diversity between big and small atolls: A case study of Yongle atoll and Lingyang reef, Xisha Islands, central of South China Sea. Biodivers. Conserv..

[23] Huang R., Yu K., Wang Y., Wang J., Mu L., Wang W. (2017). Bathymetry of the Coral Reefs of Weizhou Island Based on Multispectral Satellite Images. Remote Sens..

[24] Huang R., Yu K., Wang Y., Wang W., Mu L., Wang J. (2018). Method to design a live coral cover sensitive index for multispectral satellite images. Opt. Express.

[25] Chen G., Zhao M., Liu B., Zhang C., Liang Q. (2016). Ecological Situation of Coral Reefs in the Weizhou Island Based on Reef Check. Trop. Geogr..

[26] Hodgson G., Kiene W., Mihaly J., Liebeler J., Shuman C., Maun L. (2004). Reef Check Instruction Manual: A Guide to Reef Check Coral Reef Monitoring.

[27] Huang H., Ma B., Lian J., Yang J., Liang W. (2009). Status and conservation strategies of the coral reef in Weizhou Island, Guangxi. Trop. Geogr..

[28] English S., Wilkinson C., Baker V., English S., Wilkinson C., Baker V. (1997). Survey Manual for Tropical Marine Resources.

[29] Zhao M., Yu K., Shi Q., Yang H., Riegl B., Zhang Q., Yan H., Chen T., Liu G., Lin Z. (2016). The coral communities of Yongle atoll: Status, threats and conservation significance for coral reefs in South China Sea. Mar. Freshw. Res..

[30] Liang W., Li G.Z., Fan H.Q., Wang X., Nong H.Q., Huang H., Li X.B., Lan G.B. (2010). Species Composition and Distribution of Coral on Weizhou Island Guangxi. Guangxi Sci..

[31] Zhang Q., Shi Q., Chen G., Fong T.C.W., Wong D.C.C., Huang H., Wang H., Zhao M. (2006). Status monitoring and health assessment of Luhuitou fringing reef of Sanya, Hainan, China. Chin. Sci. Bull..

[32] Zhang L., Wei J., Fu L., Zhou C., Haffner D.G. (2015). Temporal and Spatial Variation of Nutrients and Chlorophyll-a, and Their Relationship in Pengxi River Backwater Area, Three Gorges Reservoir. Environ. Sci..

[33] D Angelo C., Wiedenmann J. (2014). Impacts of nutrient enrichment on coral reefs: New perspectives and implications for coastal management and reef survival. Curr. Opin. Env. Sust..

[34] Szmant A.M. (2002). Nutrient enrichment on coral reefs: Is it a major cause of coral reef decline?. Estuaries.

[35] Li Y., Yu K., Wang Y., Guo J., Huang X., Pei J., Luo Y. (2017). Distribution Characteristics of Surface Seawater Nutrients in Summer around Luhuitou Reef in Sanya. Trop. Geogr..

[36] Li S., Yu K. (2007). Recent development in coral reef bleaching research. Acta Ecol. Sin..

[37] Fabricius K.E. (2005). Effects of terrestrial runoff on the ecology of corals and coral reefs: review and synthesis. Mar. Pollut. Bull..

[38] Voss J.D., Richardson L.L. (2006). Nutrient enrichment enhances black band disease progression in corals. Coral Reefs.

[39] Chen T., Zheng Z., Mo S., Tang C., Zhou X. (2013). Bioerosion In Porites Corals at Weizhou Island and Its Environmental Significance. Chin. Sci. Bull..

[40] Aronson R.B., Bruno J.F., Precht W.F., Glynn P.W., Harvell C.D., Kaufman L., Rogers C.S., Shinn E.A., Valentine J.F. (2003). Causes of coral reef degradation. Science.

[41] Han L., Zheng X., Lan W., Shi X., Li T. (2015). Variations of Nutrients Concentration in Surface Seawater in Adjacent Area of Weizhou Island in Recent 10 Years. J. Appl. Oceanogr..

[42] Chen T., Zheng Z., Mo S., Zhou X., Chen T. (2013). Variation of Skeletal Extension Rate for Porites Corals around Weizhou Island in Response to Global Warming and Increase of Extreme Events. J. Trop. Oceanogr..

[43] Zhou J., Shi Q., Yu K. (2014). Exploration of factors that influence photosynthetic efficiency of symbiotic zooxanthellae of scleractinian corals in a Sanya fringing reef. J. Trop. Oceanogr..

[44] Wu H. (2016). Study on the Responses of the Growth and Photosynthetic Functions of Marine Macroalgae to Diverse Light Environments. Ph.D. Thesis.

[45] Liao Z., Yu K., Wang Y. (2016). Review on the Effect of Macroalgae on the Degeneration of Coral Reefs. Acta Ecol. Sin..

[46] Zheng Z. (2013). Studies on Water Temperature Variations in Weizhou Island & Coral Bleaching. Ph.D. Thesis.

[47] Zhang W., Zhou G.C., Gao X.Y. (2012). Analyses of Global Sea Surface Temperature Variance and Correlation. Period. Ocean Univ. Chin..

[48] Riegl B., Piller W.E. (2003). Possible refugia for reefs in times of environmental stress. Acta Diabetol..

[49] Hoegh-Guldberg O., Mumby P.J., Hooten A.J., Steneck R.S., Greenfield P., Gomez E., Harvell C.D., Sale P.F., Edwards A.J., Caldeira K. (2007). Coral Reefs Under Rapid Climate Change and Ocean Acidification. Science.

[50] Veron J., Hoegh-Guldberg O., Lenton T., Lough J., Obura D., Pearce-Kelly P., Sheppard C., Spalding M., Stafford-Smith M., Rogers A. (2009). The coral reef crisis: The critical importance of <350 ppm CO_2_. Mar. Pollut. Bull..

